# Relations of the Profession and the Public

**Published:** 1880-09

**Authors:** C. A. Brackett

**Affiliations:** Newport, R. I.


### THE DENTAL REGISTER.

VOL. XXXIV.] SEPTEMBER, 1880. [No. 9.

### RELATIONS OF THE PROFESSION AND THE PUBLIC.

BY DR. C. A. BRACKETT, D. M. D., OF NEWPORT, R. I.

GENTLEMEN :—I regret very much that I have not been able to put in better shape something in the way of a more worthy opening of the consideration of this topic I attempted the preparation of a paper, but a multiplicity of other duties forced me to lay it down as soon as commenced, and what I have to say must be presented in crude form.

Let us first think a little of the Dentist's active part in the relations. The first thought that arises is that the dentist is the servant of those for whom he works, the servant of the community in which he lives, the servant of the great public, so far as choice or necessity may bring it to him for advice and treatment, and so far as his general influence may extend. The dentist is one of that high class of servants whose knowledge concerning the work is vastly better than that of the recipient of the service; and from this fact arises much of the obligation and responsibility that rest upon him. In few callings are there more abundant opportunities for the unscrupulous and short-sighted to pursue dishonorable ways than in dentistry. Hence the right-minded practitioner is placed, by force of circumstances, on his honor, and by this must be ever guided. Great responsibilities are placed upon us; great confidences are reposed in us; and it is our part, first, to see that these responsibilities are not misplaced, and these confidences are not betrayed.

The conscientious young man preparing for the practice of dentistry, will aim to make that preparation as thorough and complete as possible. It will include many kinds of culture besides the study of strict dental science and art. At the foundation lies the physical system, and this should have such care and culture as will enable it best to accomplish its own work—such care and culture as will cause the brain, and nerves, and muscles to be healthily alert, and active, and under control. The education of the eye and hand is of surpassing importance, because they must directly accomplish the work to be done. Without manipulative dexterity of a high order a man can not hope to attain the best success in dentistry. In his mental culture the student will add to all he can learn of dentistry the study of such allied branches as can be made to serve him. Anatomy, human and comparative, histology, microscopy, physiology, surgery, general medicine, physiognomy, art expression—to say nothing of Greek, Latin, French, German and general literature—may all be profitably studied, so far as time will allow. In fact, the field of knowledge which may contribute to the excellence of the dentist's preparation is so immense, so ever-widening, there are so many experiments to be made, so many theories to be proved or disproved, so many facts to be demonstrated and established, that it is marvelous how any student or practitioner, however limited the time which he may be called upon to spend at the chair, can have any unoccupied hours.

But it is in the degree of culture to which the dentist's moral nature has been brought that much of the estimation in which he is held by patients and public must depend. Absolute freedom from every suspicion of impurity, vulgarity and coarseness, not only should there be, but the manner should always manifest that consideration, self-control, politeness, kindness, gentleness and sympathy which, while always agreeable to those with whom we have intercourse, are especially so to those under our necessary inflictions of discomfort and pain. Few men have more need of "the heart that can feel for another's woe." By this I do not mean any sickly sentimentality, but I do mean such love for humanity, and such kindly feeling, as will lead us always, while doing our duty unflinchingly, to do it with due regard for the shrinking, sensitive tissues upon which we work. If we will apply the Golden Rule to our relations with the dentist's chair, we shall be apt and alert to devise many alleviations of suffering, not at all in the way of thoroughness and excellence in our operations, but greatly to the elevation of our standing in our patient's mind and heart, if I may say it. But, understand me, all this politeness and consideration must not be put on for mere outside show and effect, for policy's sake. It must be the free, natural expression of a large heart, of true gentlemanliness; not the gentlemanliness of Chesterfield, but that gentlemanliness of which Calvert has said that perhaps it can not be better defined in one word than by "unselfishness." But the true gentleman possesses in large measure not only the negative quality of unselfishness in its restrictive sense, but also the positive one of genuine regard for the comfort and happiness and well-being of others—all of which, and much more, Calvert certainly intended to be comprehended in the definition of the true gentleman.

The dentist should be emphatically a man whose highest interest is in his calling. The Code of Ethics adopted by the American Medical Association well says: "Patients should prefer a physician whose habits of life are regular, and who is not devoted to company, pleasure, or to any pursuit incompatible with his professional obligations." While the dentist's interviews with his patients may be brought within more regular, and perhaps fewer hours than can the physician's, the statement just quoted applies with hardly lessened force to members of the former profession; or, perhaps we can change the phraseology a little, and say: The man who would achieve eminence in dentistry must have such love for his calling that all other affairs occupy at least a secondary place in his regard. The man who renders the world the greatest service in any field is the man who, with common sense, has a mission; the man who, in the depths of his heart, feels that he has a divinely-appointed work to do. Such a man avoids many things that would be distractions. He does not disregard rest, relaxation and pleasure, but he makes them his servants, to revive, to refresh, to recreate his energies, instead of by any means taxing or dulling them. The earnest practitioner makes sacrifices; but these sacrifices are only such as come in the exercise of that self-denial which a great preacher has well said means only the giving up in the present some small, trifling and transitory gratification that thereby, perhaps in the future, but surely some time, may come some greater good, more important and more enduring. Professional devotion goes far in the beginning to make the weight of these sacrifices small; and well-established habit will make them slight indeed. It is to be regretted that often we don't know when we are happy. In the confinement and weariness that sometimes we must feel, we are prone to think that if we could be free from all the cares and labors and perplexities of practice, we might have perfect enjoyment. I suppose the truth to be that, if we were forced to have just that for which we are longing, we should be of all men most miserable. Probably under no other circumstances could most of us get more happiness out of life than in the pursuit of our daily routine. Of course, we would like to have some things different from what they are; but, in the main, I have faith to believe that few combinations of circumstances could be made in this earthly life that would be better for us than those under which we are living. We want to know this, and open our eyes to see, and our hearts to feel, every day our blessedness.

Dentistry has been honored by being called a liberal profession. We should see to it that this is not a misnomer, but that our proceedings are characterized by liberality. We should be above all consideration of petty things, and labor for the accomplishment of good. Concerning pecuniary recompense for our labors, there are several points worthy of consideration. It is probably well that in the present organization of society we can not ordinarily give, and are not expected to give, our services in the apostolical way Viewed in a commercial light, the capacity of the dentist for performing operations is his stock in trade, from which he is to realize his bread and butter, and make provision for those dependent upon him, and for his own old age. This capacity, this stock, he places in the market. In each individual case it has various valuable peculiarities; in all, it is limited in amount. In the longest pay there are but a definite number of hours. In the longest lifetime there are set inexorable bounds to the working productive capacity. When it is gone, no more can be had. It is each man's privilege, then, ordinarily his duty, to sell it for the highest amount—to the highest bidder. Then, on the other hand, we are confronted with the fact that, in the present condition of things, with our present means and methods, there is a failure to preserve the teeth of the masses. The great majority of diseased teeth must go without conservative treatment, often, it is true, because such treatment is not desired, but generally because it can not be paid for. The Great Physician has said, in regard to all services, even the most trifling, which one human being can render another, "Inasmuch as ye have done it unto the least of one of these, ye have done it unto me." The thoughtful, honest man will keep these two points before him, and give to each such weight and influence as his own mind and conscience shall determine.

From the business point of view there are yet other considerations. In commercial transactions it is well understood that a low price means low quality. The converse of this statement is supposed to be generally true; unhappily, it is not always so. But it comes very naturally to be that things are valued in proportion to their cost. That which costs little is thought worth little, and given little consideration. It is the concurrent testimony of physicians that gratuitous prescriptions and free medicines do not have their directions followed with anything like the regard given to those that are paid for. Our patients should feel that the salvation of their teeth costs something, or they will not value it.

As I have said, dentistry is a liberal and an honorable calling, but the dental profession can not be liberal and honorable unless its members are so. The whole can not be better than the sum of its constituent parts. In the pursuit of a calling like this, there are proprieties to be observed and dignities to be maintained. Much of these is comprehended in the statement already made, that the dentist should be, in the truest sense, a gentleman. But there is one matter of which I wish to make special mention, because in it, and connected with it, are some of the great reproaches that rest upon us as a profession. I say us, as a profession, because the general public classes with dentists every man who chooses to call himself such, whatever his education, preparation, attainments and manner of practice may be. So, when I say dental profession now, I mean what the public calls the dental profession. I contend that the practice of advertising in the public prints, or by circulars, handbills and posters, claims of special ability and special lowness of fees—a practice in which some otherwise good men have indulged—is greatly to the discredit of the practitioner who does it, and especially derogatory to the profession of dentistry. Why? There are many reasons why. There is nothing objectionable in the dentist putting in the paper a card telling the public--those who may be in need of his services—his name, his calling and his location. Other facts concerning us and our practice will rapidly become known in any community. The publication of such a card is a modest, proper, gentlemanly thing to do; but when the self-laudatory style is adopted, the style which, for want of a better descriptive epithet, may be called "spread eagle," the gentleman is sunk in the braggart. Thereby delicate and effective instrumentation is supplanted by crude and coarse means. These means are such as must, in a measure, defeat their own ends—presuming that it is the desire of each man to work for as good a class of people as possible. The general public knows enough of such matters as we are considering to know that displayed advertising in medicine generally means quackery, and the thinking portion of it bewares. This advertising may draw a crowd, but it will assuredly keep away excellent, cultivated people who know nothing of the advertiser, and have no means of judging except from this public action of his. It is putting on the clothing of the wolf. We have high authority for avoiding not only evil, but the appearance of evil; and displayed and self-puffing advertisements in dentistry are, to the thinking man and the refined lady, the appearance of evil.

So in the matter of advertising particularly low fees. If we wish to buy a watch which shall be an exact and durable timekeeper, we don't go to the place where watches are advertised as being sold for the fewest dollars, or the fewest cents; but we go to a dealer of character and reliability, explain our want and pay his price, such a price as our own reason shows us must be put upon an article whose production requires such valuable materials and such skilled labor. If the advertisement of the watchmaker of watches for five dollars, or watches for twenty-five cents, throws suspicion upon the quality of his wares, so does the advertisement of the dentist of remarkable lowness of fees throw suspicion upon the character of his work, upon his faithfulness, upon the amount of time and labor, of brain and conscience, which he puts into his operations, and consequently upon their excellence and durability. Do not understand me as clamoring for high fees. Not at all. The dentist who works for one dollar an hour may be just as worthy a man, and just as faithful a practitioner, as he who asks ten; but I do say that a dentist whose chief claim for patronage is based on the lowness of his fees, paraded in the public prints, on large posters, signs and transparencies, is working on a narrow foundation. In its place, advertising is a great thing, one of the mightiest agencies in the world; but, notwithstanding numerous statements to the contrary, commodities are seldom voluntarily sold "less than cost."

So much for a few of the many points of relation of the dentist to those whom he serves. I have spoken particularly of these things not because they are of themselves most vitally important, but because at this time there appears to be a need of calling attention to them if we are to substantiate fully our claims to professional character. I fear that some things I have said may be misunderstood; but I am sure that I have made few statements that the right-minded practitioner and the right-minded patient, out of their own common sense, can not indorse.

Now, with your indulgence, a few words in regard to the patient's part of the relations. Of course, these are, in large measure, passive; but there are quite a number of points which should receive their active attention. One of the first of these is the selection of a dentist. They should go to a man whose character and standing are such that in him they can have confidence. While feeling it their privilege and duty to make all suggestions and express all preferences, they should be able to place themselves in his hands for such advice and such operations as his educated judgment may determine. The good patient goes to his dentist for treatment—

not "to have a job done"; and he no more thinks of asking, "Do you warrant your operations"? than a good son thinks of asking a kind and indulgent father if he will deal justly by him. To such matters as consideration of the difficulties under which the dentist labors, and faithfulness and punctuality in keeping appointments, the patient should have regard. In my own practice, I make a charge for appointments neglected without notice or some good and apparent reason, as a severe storm. In practice, too, I consider my patient's time as valuable as mine; and, if I fail to meet an appointment without sending sufficient notice, the same sum is credited on the account that would have been charged if the failure had been the patient's. Patients often make sacrifices of convenience, of pleasure, of business, in order to meet appointments with us. In domestic affairs, exigencies sometimes arise to delay a patient whose disposition to be prompt is of the best; and I come back to the dentist's duty to say that, concerning all of these things, consideration on our part should not be lacking. In our chairs, the exercise of reasonable patience and forbearance on the part of the patient is very grateful to us. In this regard, though notable exceptions occur, I am sure we should all say that, generally, we are favored quite as much as we have any reason to expect; in many cases, more so.

Probably the most important part of the patient's duty in these dental relations is to be found in the personal care given to the teeth. It is the duty of the dentist to impress upon the mind of each patient the fact that, as a rule, the preservation of the teeth in good condition depends quite as much upon constant personal care and cleanliness as upon any operations performed. Then it is the duty of the patient to spare no efforts in this personal care and cleanliness. Its neglect may lead to the failure of faithful operations, bring censure upon a worthy dentist, and reproach upon the conservative practice of our calling.

It is much to be regretted that there are many misunderstandings between patient and practitioner. As a matter of fact, men are constantly misapprehending each other, even about the commonest every-day affairs. What wonder is it, then, that we sometimes fail to be correctly understood in dental matters when honestly trying to be plain. Patients, of themselves, gain erroneous ideas, too, of the time when certain operations were performed, by whom they were performed, and on many other points. In these matters, a carefully-kept and complete record of operations is of the utmost service. Such a record is a satisfaction not only to ourselves, but it can almost invariably be so exhibited as to convince the patient of its reliability.

Mistaken conceptions arise not only between patient and practitioner, but between practitioners themselves. Mistakes will occur so long as the world endures. I suppose we can not change that fact, but we can give it practical recognition. When a person comes in and points to a filling with decay all about its borders, saying that Dr. Blank, over the way, made that filling less than a year ago, we should consider, and state to the patient, the possibility, not to say the probability, of there being some mistake, and that our brother may not be so bad as the truth of the expressed idea would indicate. Self should be the first person to hold to strict accountability. By the time we have done that, we shall probably be ready to be reasonably charitable toward others.

I have spoken of many business points in the relations of the profession and the public—of awkward and disagreeable points; but how much there is in these relations that is very pleasant! In the mutual exercise of effort, of confidence, of patience, of forbearance, of sympathy, of delicate consideration and kindly regard, how much is there to educate and develop and gratify the higher portions of our nature. I repudiate, utterly, the idea that our occupation is a harsh or hardening one. Properly followed, it affords rare opportunities for the culture of all that in us which is best—which is most akin to divinity. With self-respect, with respect and sympathy for suffering humanity, with love for the creature and love for the Creator, may the followers of our noble calling go on to more extended usefulness in yet higher fields, blessing and being blessed.—*Trans. Conn. Val. D. S.*

